# Cell-Matrix Interactions Contribute to Barrier Function in Human Colon Organoids

**DOI:** 10.3389/fmed.2022.838975

**Published:** 2022-03-10

**Authors:** James Varani, Shannon D. McClintock, Muhammad N. Aslam

**Affiliations:** The Department of Pathology, The University of Michigan Medical School, Ann Arbor, MI, United States

**Keywords:** Aquamin®, basement membrane, cell-matrix adhesion, cell-cell junction, colonoid, gut barrier, laminin, proteomics

## Abstract

The importance of cell-matrix adhesion to barrier control in the colon is unclear. The goals of the present study were to: (i) determine if disruption of colon epithelial cell interactions with the extracellular matrix alters permeability control measurement and (ii) determine if increasing the elaboration of protein components of cell-matrix adhesion complexes can mitigate the effects of cell-matrix disruption. Human colon organoids were interrogated for transepithelial electrical resistance (TEER) under control conditions and in the presence of Aquamin®, a multi-mineral product. A function-blocking antibody directed at the C-terminal region of the laminin α chain was used in parallel. The effects of Aquamin® on cell-matrix adhesion protein expression were determined in a proteomic screen and by Western blotting. Aquamin® increased the expression of multiple basement membrane, hemidesmosomal and focal adhesion proteins as well as keratin 8 and 18. TEER values were higher in the presence of Aquamin® than they were under control conditions. The blocking antibody reduced TEER values under both conditions but was most effective in the absence of Aquamin®, where expression of cell-matrix adhesion proteins was lower to begin with. These findings provide evidence that cell-matrix interactions contribute to barrier control in the colon.

## Introduction

Functional defects in the gastrointestinal tract barrier have been documented in inflammatory conditions of the bowel, including both ulcerative colitis (UC) and Crohn's Disease (1–6). Barrier defects have also been described in irritable bowel syndrome (7) and noted in celiac disease (5) and as a consequence of acute bacterial infection (8). Barrier defects have also been seen in obesity related to high-fat and high-sugar diets (9, 10) and, thus, may contribute to chronic, systemic inflammation. Finally, gastrointestinal discomfort associated with chronic environmental stress may reflect barrier dysfunction (11). In these situations, inflammatory injury to the intestinal wall contributes to barrier break-down. At the same time, however, preexisting barrier defects, leading to permeation of bacteria, bacterial products, food allergens and toxins into the mucosal wall, may promote inflammation in the gastrointestinal tract (4).

Tight junctions are the epithelial cell surface structures that mediate permeability control—at least in so far as soluble factors are concerned (12–18). Desmosomes are responsible for tissue cohesion and strength (19, 20). While not directly involved in regulating transepithelial passage of small molecules, an effective barrier in mechanically-active tissue depends on tissue cohesion. In a recent study, it was demonstrated that Aquamin®, a calcium-, magnesium-, and trace element-rich, multi-mineral product obtained from marine red algae (21), strongly up-regulated desmosomal proteins and increased the number of desmosomes in human colon tissue (obtained from normal healthy subjects and UC patients) in organoid culture but had little effect on tight junctional elements (22–24). In parallel with these desmosomal changes, tissue cohesion was increased. In addition, electrical resistance across a monolayer of organoid-derived cells was also increased (23). The same multi-mineral intervention that increased desmosomes also up-regulated expression of other moieties that contribute to the permeability barrier. Among these were cadherin family members (adherens junction components), carcinoembryonic antigen cell adhesion molecules (CEACAM), mucins and trefoils.

In the present study, we have used a proteomic screen to assess the expression of proteins involved in cell-matrix interactions in human colon organoid culture derived from either normal colon tissue or UC disease-affected tissue. Studies by other investigators have utilized immunohistochemical methods to show basement membrane defects in UC and Crohn's disease as well as in other inflammatory conditions of the bowel (25–28). While these findings suggest a role for cell-basement interactions in barrier function, how these interactions influence gastrointestinal barrier function, *per se*, has not been studied. Here it is shown that which proteins are affecting cell-basement membrane interactions through both focal adhesions and desmosomes in response to Aquamin®. Further, it has demonstrated a role of an antibody to the major cell adhesion domain in the laminin α-chain on transepithelial electrical resistance (TEER) in human colon organoid-derived monolayer. In contrast, the effect of treatment with the same antibody is evaluated on tissue cohesion/tissue strength. The findings presented here directly address the role of cell-matrix interactions in barrier function.

## Materials and Methods

### *In vitro* Intervention - Aquamin®

This is a calcium-rich, magnesium-rich, trace element-rich multi-mineral product obtained from the skeletal remains of the red marine algae, *Lithothamnion sp* (21) (Marigot Ltd, Cork, Ireland). Aquamin® contains calcium and magnesium in a molar ratio of approximately 12:1 along with measurable levels of 72 other trace minerals (essentially all of the trace elements algae fronds accumulate from the deep ocean water). The same single batch of Aquamin® Soluble that was used in the previous colon organoid studies (22–24) was used for this study. Supplementary File S1 describes the complete mineral/trace element composition of the multi-mineral product—Aquamin®.

### Anti-laminin Antibodies and Other Reagents

The known laminin heterotrimers contain a globular region in the C-terminal end of the molecule made up of five modules. Cell-binding sites are located here (29–31). A mouse monoclonal antibody (IgG1 clone) reactive against epitopes within this region (present in all of the individual α chain members) was used for functional blockade. This antibody (clone #P3H9-2; R&D Systems) has been demonstrated to detect antigen in a variety of epithelia and has been shown to inhibit cell proliferation of both rat and human epithelial cells (32). A control mouse monoclonal IgG1 immunoglobulin was used in parallel with the anti-laminin antibody for comparison. A rabbit polyclonal antibody (Invitrogen; PA5-27271) prepared against a recombinant protein fragment from the human laminin β1 chain was used in Western blotting. A monoclonal antibody recognizing a human actin epitope (Cell Signaling Technology; 5125S) was used as control.

### Organoid Culture (From Normal Colon or UC Biopsies)

Colon tissue in organoid culture was available from our previous studies (22–24). The Institutional Review Board at the University of Michigan Medical School approved the tissue collection and use protocol (IRBMED protocols: HUM00076276 and HUM00102771). Subjects provided written informed consent prior to flexible sigmoidoscopy and biopsy collection. This study was conducted according to the principles stated in the Declaration of Helsinki. For the present work, cryopreserved colon organoid samples (from healthy subjects) were put into culture and expanded over a 3-4 week period with weekly subculture during the expansion period (23). Growth medium consisted of a 50:50 mix of Advanced DMEM (Gibco) and the same base media that had been conditioned by the previous growth of L-cells engineered to provide a source of recombinant Wnt3a, R-spondin-3, and Noggin—referred to as L-WRN conditioned medium (33). The growth medium formulation also contained 100 ng/ml human recombinant epidermal growth factor (EGF) (R&D) as the major growth-supporting peptide and also contained 10 μM Y27632 (Tocris), 500 nM A83-01 (Tocris), 10μM SB202190 (Sigma), 2.5 μM CHIR99021 (Tocris), 1X B-27 without vitamin A (Invitrogen), 1 mM N-Acetyl-L-cysteine, 10 mM HEPES (Invitrogen), 2 mM Glutamax (Invitrogen), and 100 μg/ml Primocin (InvivoGen). Since L-WRN medium was supplemented with 20% fetal bovine serum, after 1:1 dilution, the final serum concentration of the growth medium was 10%. After expansion, organoids were used to assess TEER or tissue cohesion as described below. TEER assessments were carried out in either differentiation medium or in a mix of KGM Gold and growth medium.

#### Differentiation Medium

Differentiation medium consisted of a mix of Advanced DMEM and F12 media. This formulation lacked Wnt3a and R-spondin-3 but was supplemented with EGF (50 ng/ml) along with Gastrin (10 nM, Sigma), Noggin (50 ng/ml, R&D), and Y27632 (2.5 μM, Tocris). AlbuMAX® (Gibco), a lipid-rich Bovine Serum Albumin (BSA), was used as a component of the medium to replace serum. The final calcium concentration in complete differentiation medium was 1.04 mM. This medium was used as a positive control to test monolayer integrity by TEER assessment.

#### KGM Gold-Growth Medium Mix

KGM Gold is a serum-free, calcium-free medium designed for epithelial cell growth (Lonza) during experimental phase. When KGM Gold was mixed with the growth medium at a 1:4 dilution, the serum concentration decreased to 2.5% and the calcium concentration equaled to 0.25 mM (and this mix was used as a control).

### Assessment of Electrical Resistance Across the Organoid-Derived Cell Monolayer

TEER assessments were carried out in the Translational Tissue Modeling (TTML) Laboratory using a standard operating procedure developed in the TTML for organoid evaluation (34). Briefly, colon organoids (from three healthy subjects) were dissociated into small cell aggregates (<40 μm in size) and plated onto collagen IV (Sigma)-coated polyethylene terephthalate filters (0.4 μm pore size, 0.33 cm^2^, in transwell filter support, Costar) at 200,000 individual organoids per well in growth medium. After seeding in growth medium, organoids were allowed to attach to the transwell insert filters and incubated without further treatment for 1 day. Then growth medium was replaced with either differentiation medium alone (for initial assessment) or with the KGM Gold-growth medium mix with or without Aquamin®. When Aquamin® was included, it was added at 0.51 mg/ml; an amount to bring the final calcium concentration to 1.5 mM.

The function-blocking anti-laminin antibody (an antibody to the major cell adhesion domain in the laminin α-chain) was included at the start of the treatment period at 25 μg/ml. Fresh culture medium and antibody were provided every 2 days during the assay period. A control mouse IgG was used at the same concentration for comparison. Electrical resistance values were determined using an epithelial volt/ohm meter (EVOM2, World Precision Instruments) and STX2 series chopstick electrodes as described previously (23).

### Histochemical Staining and Light Microscopy

After finishing electrical resistance measurements, transwell insert filters with organoid-derived monolayer cells still attached were prepared for light microscopy. The transwell insert filters were fixed for 1 h in 10% buffered formalin. Following this, insert filters were paraffin-embedded, sectioned and stained with hematoxylin and eosin. The stained specimens were visualized by light microscopy. Slides were digitally scanned using the Aperio AT2 brightfield whole slide scanner (Leica Biosystems) at a resolution of 0.5 μm per pixel with 20X objective. Quantitation was performed using Aperio ImageScope by measuring the gap between the epithelial layer and the transwell insert membrane at 20 × magnification.

### Western Blotting

After finishing electrical resistance measurements on Day 3, organoid-derived monolayer cells were harvested for protein. Briefly, insert wells were washed gently with PBS, then subjected to extraction using RIPA buffer (89901; Thermo Scientific). Organoid-derived monolayer cells were lysed by repetitive pipetting in the buffer, followed by incubation for 10 min on ice. Non-soluble cellular debris was removed by centrifugation at 14,000 x g for 10 min and protein was quantified using a BCA assay (23227; Pierce). Samples were heated for 10 min at 70°C in NuPage LDS sample buffer and then run on 3-8% Tris-Acetate gels using NuPage MOPS running buffer under reducing conditions. Proteins were then transferred onto nitrocellulose membranes, blocked with 5% non-fat dry milk and probed with the primary and appropriate secondary antibodies. Secondary antibodies were used at 1:5,000 for all membranes. β-actin was used as a loading control in each assay. SuperSignal WestPico Plus (34577; Thermo Scientific) detection reagent was used and bands were visualized by exposing the membranes on CL-XPosure Film (34090; Thermo Scientific) and developing the films using Konica Minolta SRX-101A. Relative band density was determined using ImageJ gel analysis tools.

### Confocal Fluorescence Microscopy

After finishing electrical resistance measurements, some of the transwell insert filters were prepared for confocal fluorescence microscopy and stained with an antibody to occludin for the purpose of visualizing the cell layer. The filters with cells still attached were fixed for 15 min at −20°C in methanol. They were then washed three times in PBS before blocking in 3% BSA (A8806; Sigma) in PBS for 1 h. Following this, cells were stained with an antibody to occludin (331594; Invitrogen) 1:400 for 1 h in 1% BSA in PBS. Stained cells were rinsed three times (5 min each) in PBS, stained with DAPI for 5 min to identify nuclei and washed an additional three times with PBS. Finally, the filters with cells still attached were gently cut from the transwell inserts and mounted apical side up on Superfrost Plus glass slides (Fisher Scientific, Pittsburgh, PA) with Prolong Gold (P36930; Life Technologies Molecular Probes). The stained specimens were visualized and imaged with a Leica Inverted SP5X Confocal Microscope System (University of Michigan Medical School Biomedical Research Core Facility).

### Organoid Cohesion Assay

Organoid cohesion was assessed by employing healthy colon-derived organoids from three subjects as described previously (23). Briefly, after establishment and culture expansion, healthy colon organoids were incubated in KGM Gold-growth medium with or without the same anti-laminin antibody (25 μg/ml) as described above. Treatment was for seven days with fresh medium and antibody added at days 2 and 4. Over the course of the 7-day treatment period, individual organoids increased in size. At the end of the incubation period, phase-contrast microscopy (Hoffman Modulation Contrast—Olympus IX70 with a DP71 digital camera) was used to capture images in order to measure the size of multiple individual organoids (53-104 individual organoids per condition). Then organoids were separated from the Matrigel and fragmented with mechanical force alone by pipetting the entire pellet 30x through an uncut 200 microliter pipet tip. After washing 3x in PBS, organoids were re-cultured in fresh Matrigel. One day after establishment, multiple organoids were again examined under phase-contrast microscopy and sized. For both pre-harvest and post-harvest samples, phase-contrast images were analyzed using area measurements in Adobe Photoshop (CC version 19.1.5). Average organoid size-reduction (i.e., the difference in organoid size between pre- and post-harvest) was determined by dividing the average post-harvest surface area by the average pre-harvest area.

### Differential Proteomic Analysis

Proteomic assessment was conducted at the Proteomics Resource Facility (PRF) in the Department of Pathology at the University of Michigan using mass spectrometry (MS)-based tandem mass tag (TMT) analysis (ThermoFisher Scientific). The complete details for the experimental conditions, protocols and analysis methodology can be found in previously published reports (22, 24). Briefly, colon organoids (normal healthy subjects and subjects with UC) were exposed to 2mM EDTA for 15 min to dissolve and completely remove Matrigel and then exposed to Radioimmuno-precipitation assay (RIPA)—lysis and extraction buffer (Thermo Scientific, Rockford, IL) for protein isolation. Fifty micrograms of organoid protein from each condition were digested with trypsin and individually labeled with isobaric mass tags. Labeled peptides were fractionated using 2D-LC (basic pH reverse phase separation followed by acidic pH reverse-phase) and analyzed on a high-resolution, tribrid mass spectrometer (Orbitrap Fusion Tribrid, ThermoFisher Scientific) using conditions optimized in the PRF. MultiNotch MS3 was employed to obtain accurate quantitation of the identified proteins/peptides. Data analysis involved peptide filtering to retain only those that passed ≤ 2% false discovery rate (FDR) threshold of detection. Quantitation was performed using high-quality MS3 spectra. Differential protein expression values (fold-change) for proteins of interest in each treatment group were compared to protein values of the respective control group. Proteins were identified using Universal Protein Resource (UniProt) databases (Uniprot.org). Reactome version 78—a pathway analysis database was used to recognize associated pathways for species “*Homo sapiens*” (reactome.org) by providing the entities detected in the proteomic data sets (both from normal and UC data sets). Reactome is a curated and peer-reviewed database of pathways and reactions in human biology. Reactome database identifies possible reactions with all annotated proteins present and active simultaneously in a cell. Pathway over-representation analysis is performed by overlaying an experimental dataset on these annotations (35). Additionally, STRING database—v11.5 (string-db.org) was utilized to conduct enrichment analyses and to identify protein-protein interactions among the proteins. For proteomic enrichment analysis, STRING employs Gene Ontology (GO) knowledgebase and provide information related to molecular functions, biological processes and cellular components involved.

For the purpose of the present study, we accessed two existing data sets—one generated from colon organoids of four healthy subjects and the other generated from colon organoid tissue of three ulcerative colitis patients in remission. In each case, organoids grown in the KGM Gold-growth medium mix were compared to organoids grown in the same medium supplemented with Aquamin® at levels providing 1.5-3.0 mM calcium. Protein expression levels with Aquamin® were compared to protein-expression levels in the control to obtain fold-change ratios for individual proteins of interest with each subject separately. Following this, data from individual subjects were merged and analyzed as groups (*n* = 4 healthy and *n* = 3 UC in remission). For comparison purposes, the data presented here include only the maximum response. The complete proteomics data sets are available at the ProteomeXchange Consortium via the PRIDE partner repository with the dataset identifier PXD020244 (for UC derived colon organoids) and identifier PXD026923 (for normal colon organoids).

### Statistical Analysis

Means and standard deviations were obtained for discrete values obtained in the TEER assessment and cohesion assays as well as from expression level changes for individual proteins in proteomic assessment). Data generated in this way were analyzed by ANOVA followed by paired *t*-test (two-tailed) for comparison using GraphPad Prism version 8.3. For the pathways analysis, the significant data were based on the overrepresentation analysis (hypergeometric distribution) using Reactome database. A binomial test was used to calculate the probability for each result, and the *p*-values were corrected for the multiple testing (Benjamini–Hochberg procedure) that arose from evaluating the submitted list of identifiers against every pathway. A high-level of FDR stringency (<1%) was used and the whole genome statistical background was assumed for STRING analysis. A *p*-value < 0.05 was considered significant.

## Results

### Aquamin® Up-Regulates Basement Membrane Components, Proteins Associated With Hemidesmosome Formation and Keratins

**Figure 1:**
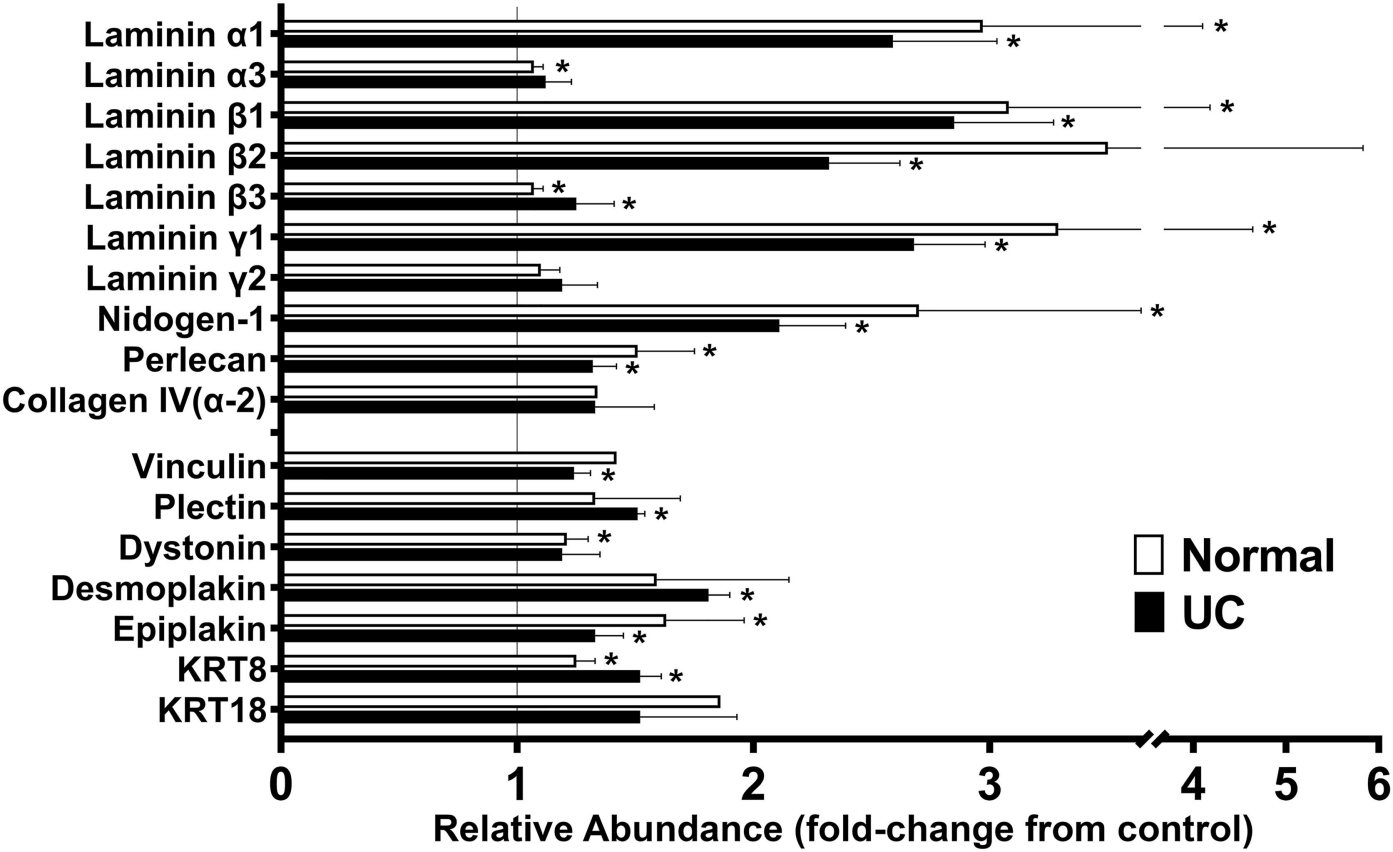
Effects of Aquamin® on expression of proteins involved in cell-matrix interactions: Proteomic screen. Organoid cultures were established with colon biopsies from four healthy subjects and three with ulcerative colitis in remission. Organoids were cultured in the presence or absence of Aquamin® as described in the Materials and Methods section. At the end of the incubation period, protein was isolated from the cultures and assessed in the proteomic screen. Values from respective controls were set at 1.0 and the values from Aquamin®-treated samples reflect a ratio (increase or decrease fold-change) relative to respective control. Values shown reflect mean (±SD) fold-change from respective control in colon organoids from normal subjects (*N* = 4) and subjects with UC in remission (*N* = 3). Methods for generating the data sets were described in detail in past reports (22, 24). Data for individual proteins were compared for statistical differences using the student *t*-test. Asterisk (*) indicates a difference from respective control at *p* < 0.05.

Findings from the proteomic assessment based on data from four healthy subjects and data from three subjects with UC are shown in Figure 1. In both data sets (assessed independently), strong up-regulation of several laminin chains (α1, β1, β2, and γ1) (components of laminin 111 and 121) along with nidogen-1, the basement membrane-specific heparin sulfate proteoglycan (HSPG-2, perlecan) and one of the chains of type IV collagen (α2) was seen in response to Aquamin®, regardless of the tissue type. These proteins are the major constituents of the basement membrane (36, 37). They mediate cell-matrix attachments (focal adhesions) in epithelial cells (30, 31, 36, 37). Also detected in the proteomic analysis (Figure 1) were laminin α3, β3, and γ2 chains (components of laminin 332 or laminin-5 in the older terminology). This laminin isoform is a major component of hemidesmosomes (38, 39). While these laminin chains did not demonstrate an increase in response to Aquamin® in colon organoids, several other hemidesmosomal proteins were detected, and a subset of these (dystonin, plectin, desmoplakin, and epiplakin) were also increased by Aquamin® as compared to control (Figure 1). The plakins are critical linkers between laminin in the hemidesmosomes and intermediate filaments (40, 41). Similarly, additional hemidesmosomal components (BP180 or Collagen Type XVII α1 Chain, and CD151) were also detected in both datasets. The abundance ratios for BP180 (1.14 ± 0.19-fold in normal vs. 1.21 ± 0-fold in UC) and CD151 (0.94 ± 0.07-fold in normal vs. 0.96 ± 0.06-fold in UC) did not increase with Aquamin®. In addition, three proteins that serve as connectors between focal adhesions and the actin cytoskeleton (talin, vinculin and α-actinin) were detected. Vinculin was modestly up-regulated in both data sets (Figure 1).

While this study did not address differentiation-related proteins, *per se*, since we have previously reported on this (22, 24), we noted that keratin 8 and keratin 18 (components of intermediate filaments in gastrointestinal epithelial cells) (42) were increased in response to Aquamin® (Figure 1). With keratin 8, expression was increased 1.25 ± 0.01-fold and 1.52 ± 0.09-fold in the normal and UC data sets, respectively. With keratin 18, values were 1.52 ± 0.41-fold and 1.86 ± 0.00-fold. Of interest, recent studies have demonstrated that mutations in Keratin 8/18 in colonic epithelial cells are associated with loss of permeability control in inflammatory bowel disease (43). Similarly, acute bowel inflammation has been shown to reduce Keratin 8/18 expression; levels were restored upon improvement in disease status as assessed by both clinical and endoscopic parameters (44).

In addition to the findings presented above, other proteins of interest were searched for in the protein screen. Subunits of laminin-binding integrins (α3, α6, β1, and β4) (45) were present, but not significantly altered (ranged from 0.90 to 0.96-fold-change) with Aquamin® as compared to control (not shown). Among other moieties that have been reported to interact with laminin, both dystroglycan and syndecan were slightly down-regulated, sulfatide was unchanged and oncofetal antigen/immature laminin receptor OFA(iLRP)/67-kD laminin receptor was not detected.

As part of the analysis, we searched for the pathways associated with the proteins presented in Figure 1 using Reactome. The top 20 pathways with the involved entities are presented in Table 1. Laminin interactions, extracellular matrix organization and type I hemidesmosome assembly were among the top pathways (Table 1). To check the protein-protein interaction (PPI) of the moieties shown in Figure 1, we used the STRING database, and the PPI enrichment *p*-value was < 1.0 × 10^−16^. Supplementary Figure 1 showed these strong protein-protein interactions among these proteins. Lastly, we have shown the GO-based enrichment data in Supplementary Table 1. There were 38 biological processes, 7 molecular functions, and 31 cellular components involved based on these annotations (Supplementary Table 1). These data further demonstrated the involvement of these proteins in various cell-cell and cell-matrix adhesion-related processes as suggested by the pathways analysis. Basement membrane, laminin complex, extracellular region, extracellular space, anchoring junction and extracellular exosome are some of the top cellular components involved (Supplementary Table 1).

**Table 1:** Top pathways associated with the proteins presented in Figure 1.

**Pathway name**	**Entities p-value**	**Entities FDR**	**Mapped entities**
Laminin interactions	1.11 × 10^−16^	5.55 × 10^−15^	COL4A2;LAMA1;LAMA3;LAMB1;LAMB2;LAMB3; LAMC1;LAMC2;HSPG2;NID1
Non-integrin membrane-ECM interactions	1.11 × 10^−16^	5.55 × 10^−15^	COL4A2;LAMA1;LAMA3;LAMB1;LAMB2;LAMB3; LAMC1;LAMC2;HSPG2
Extracellular matrix organization	2.22 × 10^−15^	7.33 × 10^−14^	COL4A2;DST;LAMA1;LAMA3;LAMB1;LAMB2;LAMB3; LAMC1;LAMC2;HSPG2;NID1;PLEC
MET activates PTK2 signaling	3.00 × 10^−14^	7.49 × 10^−13^	LAMA1;LAMA3;LAMB1;LAMB2;LAMB3;LAMC1;LAMC2
MET promotes cell motility	2.64 × 10^−13^	5.29 × 10^−12^	LAMA1;LAMA3;LAMB1;LAMB2;LAMB3;LAMC1;LAMC2
Type I hemidesmosome assembly	7.62 × 10^−12^	1.22 × 10^−10^	DST;LAMA3;LAMB3;LAMC2;PLEC
ECM proteoglycans	1.93 × 10^−11^	2.70 × 10^−10^	COL4A2;LAMA1;LAMA3;LAMB1;LAMB2;LAMC1; HSPG2
Degradation of the extracellular matrix	2.27 × 10^−11^	2.73 × 10^−10^	COL4A2;LAMA3;LAMB1;LAMB2;LAMB3;LAMC1; LAMC2;NID1;HSPG2
Signaling by MET	2.75 × 10^−11^	3.03 × 10^−10^	LAMA1;LAMA3;LAMB1;LAMB2;LAMB3;LAMC1;LAMC2
Assembly of collagen fibrils and other multimeric structures	4.49 × 10^−10^	4.49 × 10^−9^	COL4A2;DST;LAMA3;LAMB3;LAMC2;PLEC
Collagen formation	4.51 × 10^−9^	4.06 × 10^−8^	COL4A2;DST;LAMA3;LAMB3;LAMC2;PLEC
Anchoring fibril formation	9.57 × 10^−9^	7.66 × 10^−8^	COL4A2;LAMA3;LAMB3;LAMC2
Cell junction organization	2.88 × 10^−7^	2.02 × 10^−6^	DST;LAMA3;LAMB3;LAMC2;PLEC
Signaling by receptor tyrosine kinases	7.83 × 10^−7^	5.48 × 10^−6^	COL4A2;LAMA1;LAMA3;LAMB1;LAMB2;LAMB3; LAMC1;LAMC2
Cell-cell communication	1.57 × 10^−6^	9.39 × 10^−6^	DST;LAMA3;LAMB3;LAMC2;PLEC
Signal transduction	0.001	0.003	COL4A2;DSP;DST;LAMA1;LAMA3;LAMB1;LAMB2; LAMB3;LAMC1;LAMC2;VCL
Post-translational protein phosphorylation	0.001	0.003	LAMB1;LAMB2;LAMC1
L1CAM interactions	0.001	0.004	LAMA1;LAMB1;LAMB2;LAMC1
Formation of the cornified envelope	0.001	0.004	DSP;KRT8;KRT18
Keratinization	0.005	0.014	DSP;KRT8;KRT18

*The pathway analysis was conducted by Reactome database (v78) for species “Homo sapiens” employing the entities presented in Figure 1. These significant data (with a p-value/FDR < 0.05) are based on the overrepresentation analysis (hypergeometric distribution). A binomial test is used to calculate the probability for each result, and the p-values are corrected for the multiple testing (Benjamini–Hochberg procedure) that arises from evaluating the submitted list of identifiers against every pathway. FDR, False discovery rate*.

**Figure 2:**
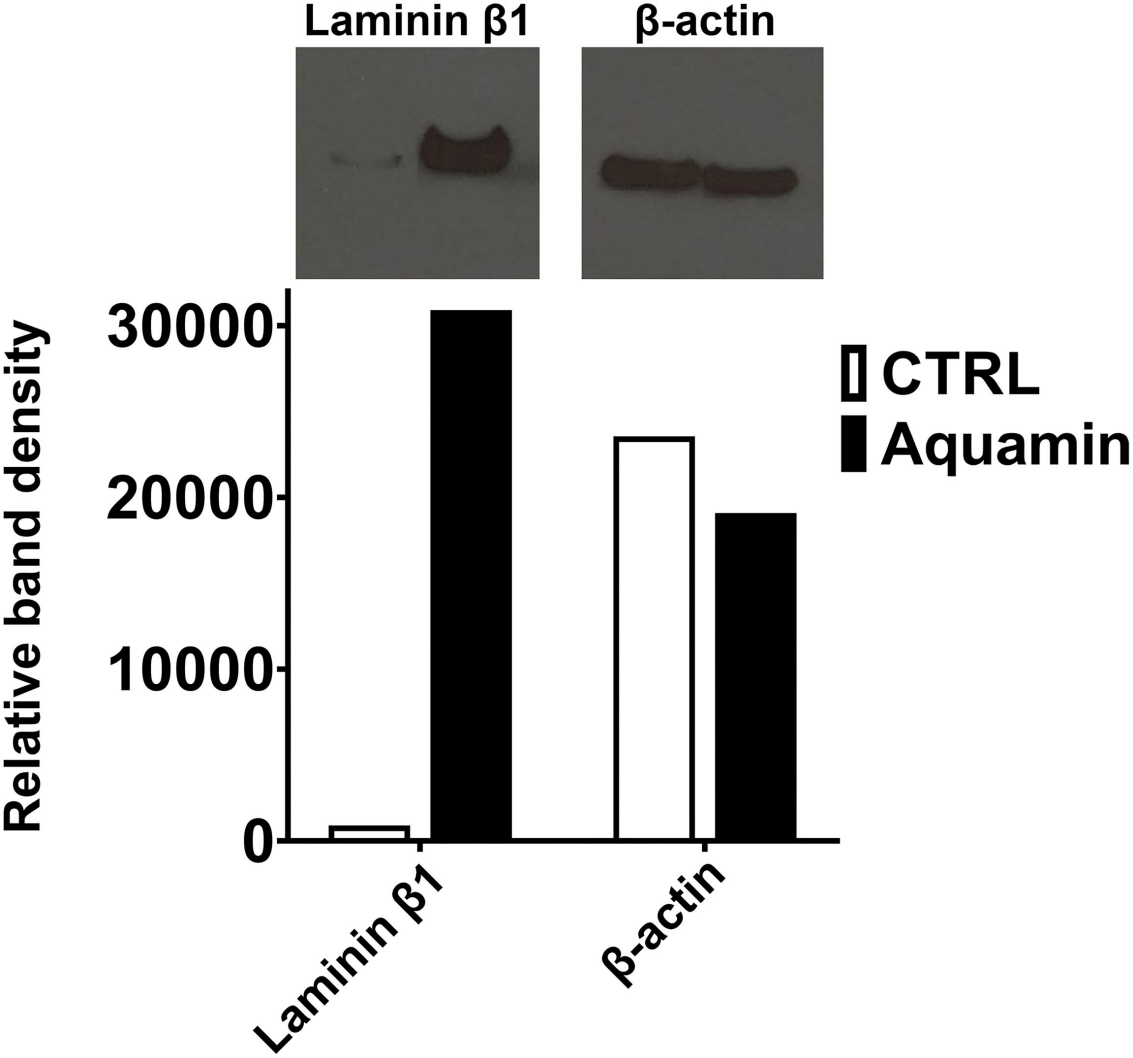
Effects of Aquamin® on expression of Laminin β1: Western blotting. Protein isolated from control and Aquamin®-treated (healthy normal subjects) colon organoid-derived monolayer cells was assessed for laminin β1 expression by Western blotting as described in the Materials and Methods section. 10 μg of protein from each condition was used. β-actin was assessed in parallel (as a loading control). Band quantitation was done using ImageJ software. Relative band density is presented for laminin β1 and β-actin.

Western blotting with an antibody to the laminin β1 chain (most highly up-regulated of all the laminin chains detected in the proteomic screen) was used to confirm laminin up-regulation. Figure 2 shows the remarkable increase in laminin β1 expression in Aquamin®-treated organoids as compared to control. The complete film along with the nitrocellulose blot are presented in the Supplementary Figure 2.

### TEER Values in Cell Monolayers Established From Organoids: Effects of Aquamin® and Anti-laminin Treatment

**Figure 3:**
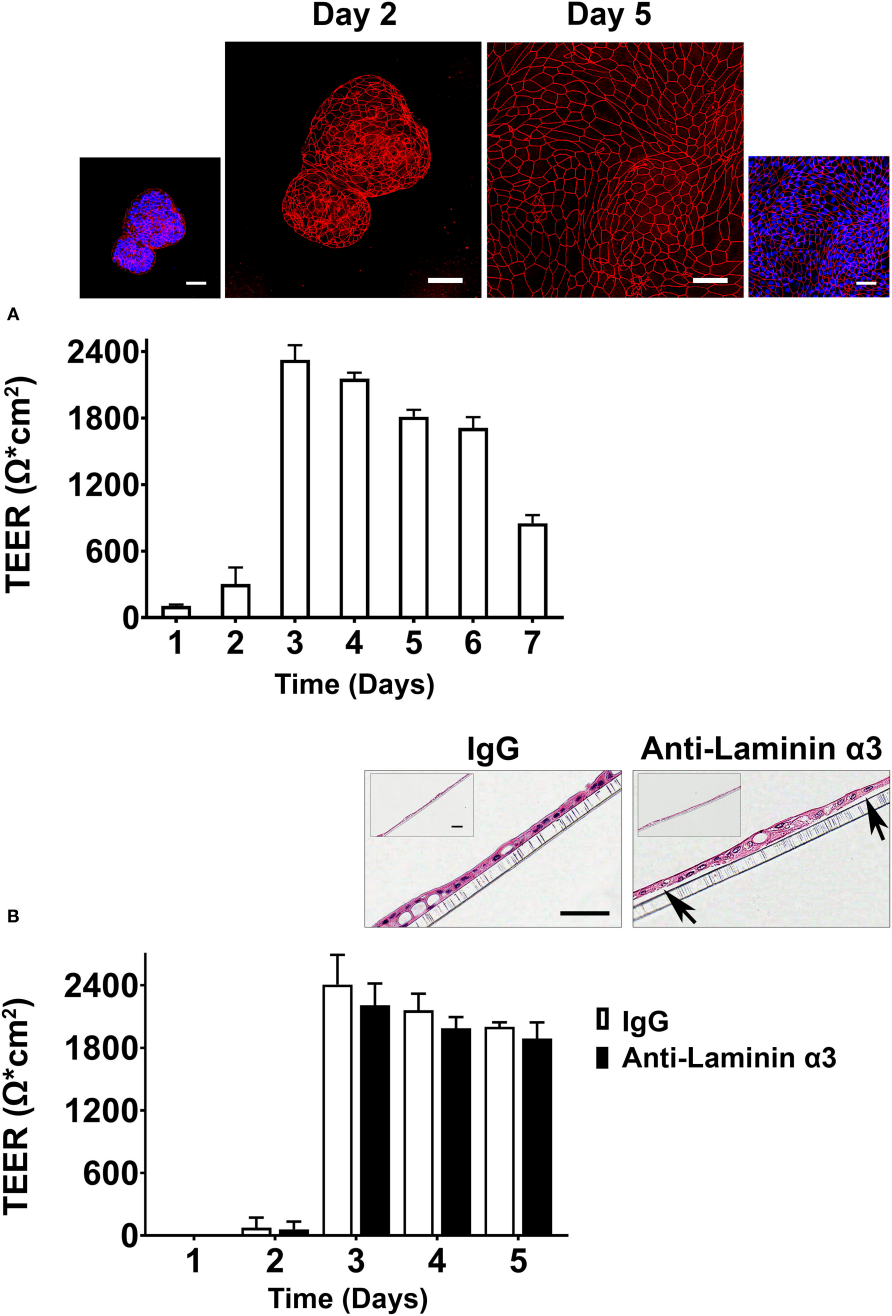
Transepithelial electrical resistance in differentiation medium (Preliminary assessment). **(A)** Time-dependent changes in TEER values. Values shown are means and standard deviations based on four separate experiments with four samples (individual transwell insert filters seeded with healthy colon organoid-derived monolayer cells) per data point at each time-point in each experiment. Insert: Confocal fluorescent microscopic (max-projected) images of organoids and organoid-derived cells on transwell inserts stained after the day-2 and day-5 readings with antibody to occludin and with a combination of antibody to occludin and DAPI. Scale bars = 50 μm. **(B)** Effects of anti-laminin antibody on TEER values. Values shown are means and standard deviations based on two separate experiments with 4 samples (individual transwell insert filters seeded with healthy colon organoid-derived monolayer cells) per data point at each time-point in each experiment. Insert: hematoxylin and eosin-stained images of the cell monolayers still attached to the transwell inserts from IgG-treated and anti-laminin-treated wells. Arrows in the anti-laminin-treated image show areas where cell detachment from the underlying transwell insert was visible. Scale bar = 100 μm (small) and 50 μm (Large).

Preliminary studies were carried out (following the standard operating procedure) in the TTML. For these studies, organoids were plated on transwell insert filters in growth medium. One day later, growth medium was replaced with a formulation optimized in the TTML for assessing electrical resistance (34). This formulation, referred to as differentiation medium, was described in the Materials and Methods section. TEER values were determined daily beginning on the next day. Results are shown in Figure 3. Figure 3A demonstrates that under conditions optimized to promote electrical resistance, TEER values were low during the first 2 days after treatment, rose precipitously at day-3, remained elevated through day-6 (except with a slight decrease every day) and declined thereafter. A combination of antibody to occludin (tight junctional protein) and DAPI (nuclear stain) was used to illuminate organoids and cell outgrowth from the organoids on the transwell filters at day-2 and day-5. As shown in the inserts in Figure 3A, intact cell-cell borders could be seen between cells in the organoids, themselves, by day-2. However, cell outgrowth from the organoids did not completely cover the transwell insert filter surface at this time (accounting for the lack of electrical resistance). Coverage of the filter surface was complete by day-5. The effects of the function-blocking antibody—anti-laminin α3 (25 μg/ml) on electrical resistance in differentiation medium are shown in Figure 3B. A modest decrease in TEER values was observed at days-3,−4, and−5 (9-17% decrease; not statistically significant). Lower antibody concentrations were not effective.

In parallel, electrical resistance was assessed in the KGM Gold-growth medium mix. Similar to what was observed in differentiation medium, TEER values were low on day 1 and day 2 (<100 Ω x cm^2^) but rose sharply such that maximum values were observed on day-3 (1,700-1,900 Ω x cm^2^), depending on experiment. Values remained elevated through day-5 and then fell (not shown).

At the completion of TEER assessment (on day-3) in differentiation medium, transwell insert filters with cells still attached were fixed in 10% buffered formalin, stained with hematoxylin and eosin and examined at the light microscopic level (Figure 3B insert). It can be seen that under control conditions (IgG-treated cells) or in the same medium with anti-laminin antibody, the filter surface was covered with a complete monolayer of cells. However, in the presence of the anti-laminin antibody, focal areas where cells had detached from the underlying substrate could be observed. In these areas, cell-cell attachments remained intact such the structure had the appearance of a tiny blister. When these visible gaps in the detached monolayer were digitally quantified, the gaps decreased from 16 ± 18 μm in the presence of anti-laminin antibody to 5 ± 7 μm under control conditions.

Based on the outcome of the preliminary studies, KGM Gold-growth medium was used in subsequent experiments. Anti-laminin antibody was included at a final concentration of 25 μg/ml and electrical resistance was determined at day-3.

**Figure 4:**
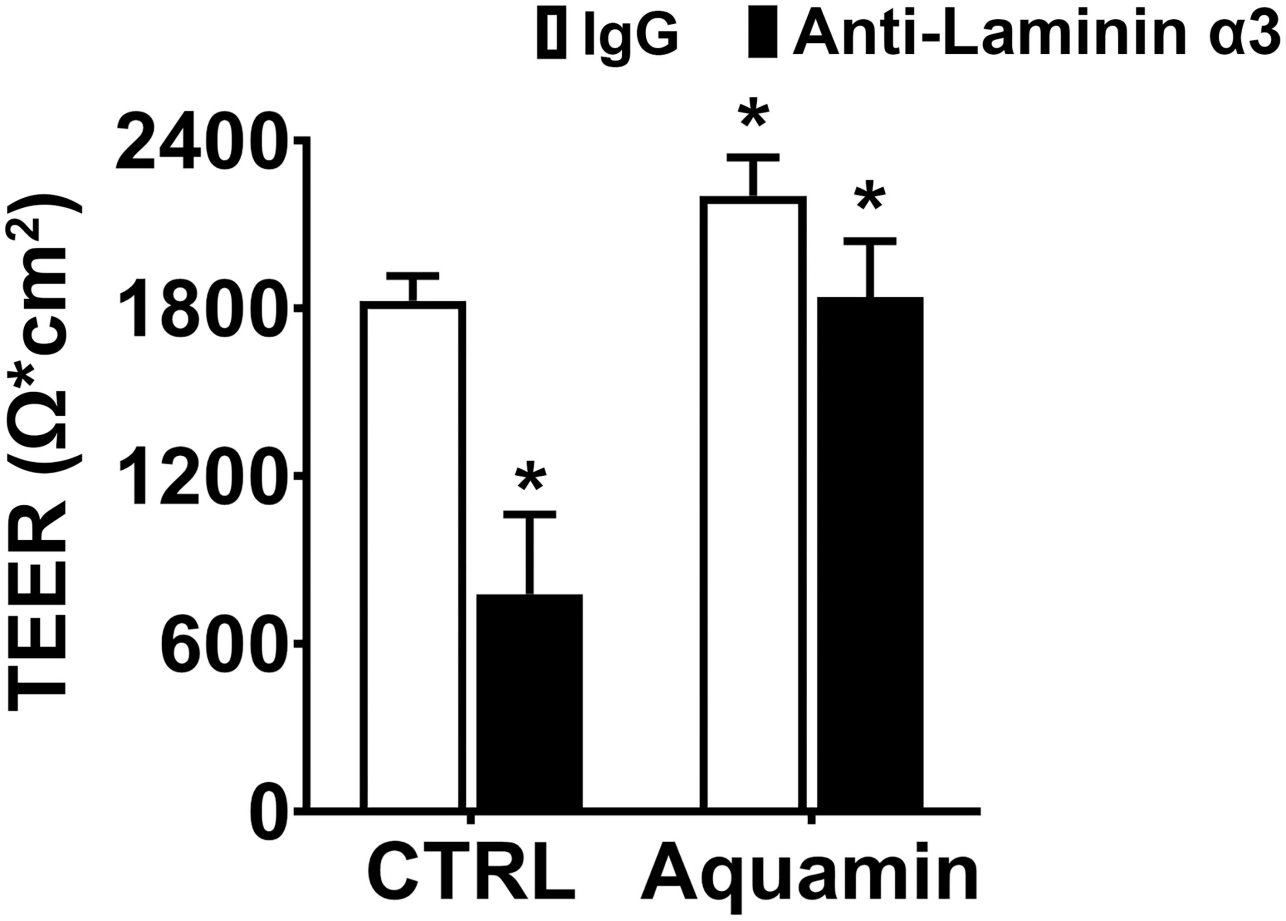
Transepithelial electrical resistance in KGM Gold-growth medium with or without Aquamin® and with or without anti-laminin antibody. TEER values shown are means and standard deviations based on three separate experiments with four samples (individual transwell insert filters seeded with healthy colon organoid-derived monolayer cells) per data point in each experiment. Data were compared for statistical differences using ANOVA followed by unpaired-group comparisons. Asterisk (*) above the open Aquamin® bar indicates a difference from control at *p* < 0.05. Asterisks (*) above the closed bars indicates difference from respective IgG control at *p* < 0.05.

Following the preliminary studies described above, human colon organoids were plated on transwell filters in growth medium. One day later, growth medium was replaced with the KGM Gold-growth medium mix (0.25 mM calcium; final concentration). In some wells, Aquamin® was added to bring the final calcium level to 1.5 mM and provide the additional trace elements that make up the marine algae product. Electrical resistance across the cell layer was assessed as described above on day-3). In the unsupplemented KGM Gold-growth medium mix, a TEER value of 1,828 Ω x cm^2^ was achieved as compared to 2,325 Ω x cm^2^ in differentiation medium (21.5% decrease) while the TEER value in Aquamin® supplemented medium (2,214 Ω x cm^2^) was virtually identical to that seen in differentiation medium (compare values in Figure 4 with those in Figure 3A—Day 3). Figure 4 also shows the effects of anti-laminin treatment on TEER values in the two conditions. In Aquamin-supplemented medium, TEER values were reduced by 16% with anti-laminin. This is comparable to what was seen in differentiation medium (compare values to those in Figure 3B). In unsupplemented KGM Gold-growth medium, where TEER values were lower to begin, the inclusion of anti-laminin antibody further reduced TEER values to 787 ± 288 Ω x cm^2^ (57% decrease).

### Effects of Anti-laminin Antibody on Organoid Cohesion

**Figure 5:**
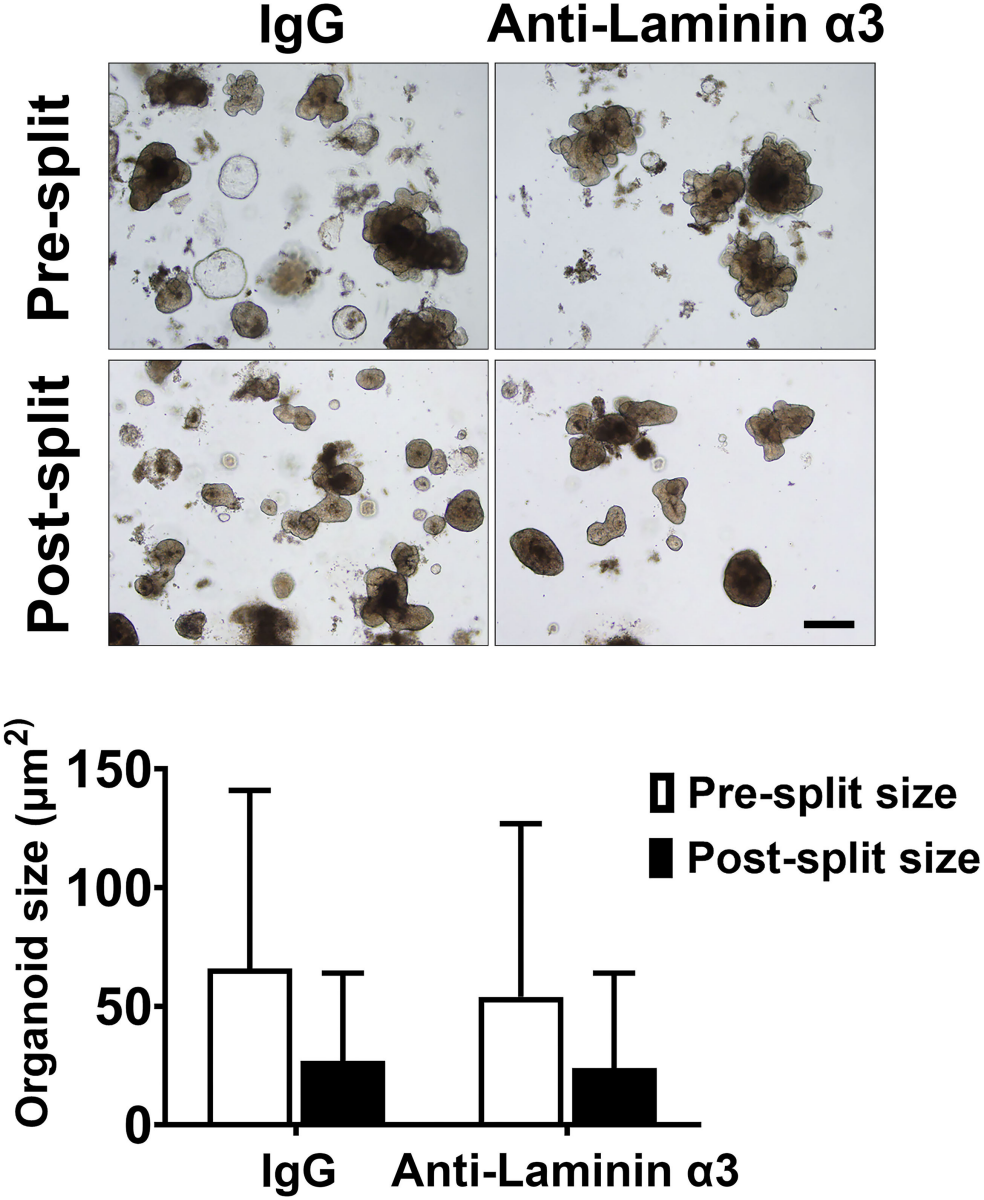
Colon organoid cohesion in KGM Gold-growth medium mix: Effect of anti-laminin antibody. Colon organoids were maintained for 7 days in KGM Gold-growth medium with either IgG or anti-laminin. At the end of the incubation period, organoid cohesion was assessed as described in the Materials and Methods section. Values shown represent the change in organoid size (i.e., mean surface area ± SD of individual colon organoids based on two separate experiments with a minimum of 53-104 colon or-ganoids assessed individually per treatment group in both pre- and post-harvest cultures. Data were compared for statistical differences using ANOVA followed by unpaired-group comparisons. While the decrease in organoid size between post-harvest and pre-harvest organoids were statistically significant with either IgG or anti-laminin, the differences between anti-laminin and IgG were not different. Inset: Representative examples of organoid appearance immediately prior to harvest (upper) and 1 day after the harvested organoids had been reestablished in culture. Scale bar = 200 μm.

In our previous study, we demonstrated that treatment of human colon organoids with Aquamin® increased organoid cohesion in parallel with TEER values. Specifically, organoids maintained in KGM Gold-growth medium without Aquamin® fragmented into much smaller pieces than did organoids grown in the presence of Aquamin® and subjected to the same mechanical disruption protocol (23). We attributed increased cohesion in the presence of Aquamin® to the increase in desmosomes seen in parallel. This does not, of course, rule out the possible contribution of other adhesive interactions. To determine whether interactions involving laminin contributed to intra-organoid cohesion, colon organoids were maintained for 1 week in KGM Gold-growth medium with either IgG or the same anti-laminin antibody that reduced TEER values. At the end of the incubation period, cohesion was assessed as described in Methods and in our previous study (23). No detectable antibody effect on organoid cohesion was seen. Specifically, there was no difference between IgG-treated and anti-laminin-treated organoids in average organoid size after harvest and fragmentation (i.e., post- to pre-harvest ratio). This was 0.45 and 0.46 with IgG and anti-laminin antibody, respectively (Figure 5).

## Discussion

Most studies of barrier dysfunction in the gastrointestinal tract have focused on the structural components that regulate cell-cell interactions (i.e., desmosomes and, especially, tight junctions) (16–19), but basement membrane disruptions are also commonly observed (25–28). Experimental animal models of colitis, likewise, demonstrate basement membrane disruptions in inflamed colonic tissue (28, 46). In all of these settings, a loss or reduction in laminin immunoreactivity is commonly observed (25–28), although altered distribution of laminin forms has been reported as well, with some forms actually increasing (27). Laminin is not unique in being altered in inflammatory bowel conditions. Basement membrane collagens including type IV have been reported to be increased in inflamed bowel (28). Together, these past findings provide a picture of widespread cell-matrix disruption in the context of the inflamed colon. Although these changes are thought to be a consequence of the inflammatory process, anomalies have been noted in some patients with inflammatory bowel disease in the absence of acute tissue damage (4). Thus, preexisting basement membrane irregularities may contribute to inflammation, and not simply be the consequence of tissue injury. In support of this, a murine model in which laminin α-chain was overexpressed showed a decreased sensitivity to chemical-induced colitis (28). In another model, hemidesmosome disruption promoted colitis (46) in genetically manipulated animals.

**Figure 6:**
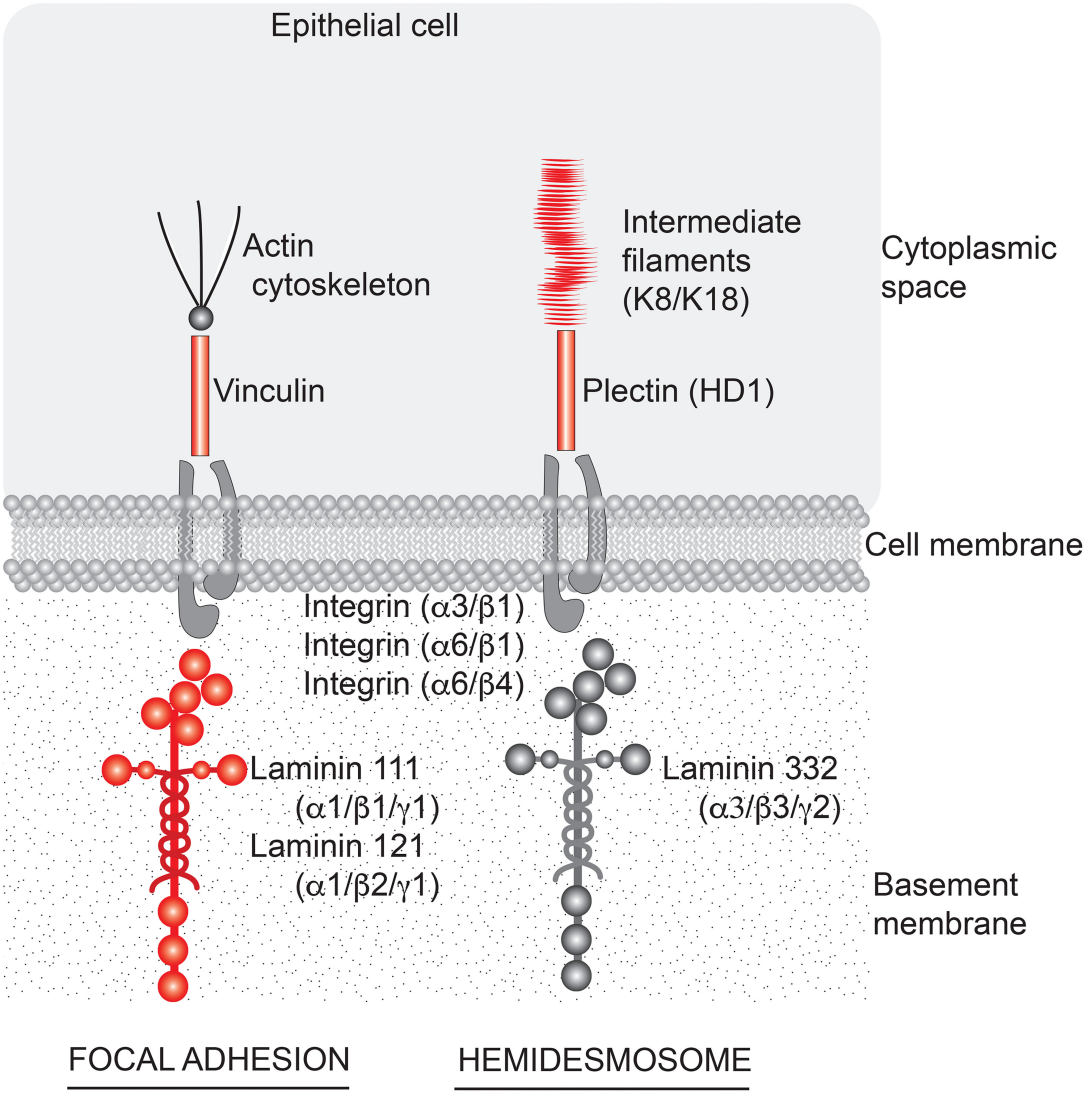
Aquamin®-responsive cell-matrix adhesion structures in the colon. A cartoon depicting structures important to cell-matrix adhesion in the colon and components of those structures that are responsive to Aquamin® (shown in red). Based on the profile of proteins that are induced by Aquamin®, cell-matrix adhesion through both focal adhesions and hemidesmosomes could be affected. Laminin-binding integrins did not alter with Aquamin®.

Regardless of whether preexisting barrier defects in the gastrointestinal tract promote bowel inflammation or are simply the consequence of inflammation, improvement in barrier structure/function would seem to be of value. The findings presented here demonstrate that interfering with cell-basement membrane interactions reduces electrical resistance across the cell layer (a measure of permeability control) without a major effect on tissue cohesion in human colon organoid culture. Our findings also demonstrate that treating colon organoids with a multi-mineral supplement increases the elaboration of basement membrane proteins and hemidesmosomal/intermediate filament components while partially mitigating the consequences of interfering with cell-basement membrane interactions. As summarized graphically in the cartoon (Figure 6), the basement membrane, hemidesmosomal and intermediate filament proteins that are responsive to Aquamin® treatment could be expected to have an effect on both focal adhesions and hemidesmosomes (47). In our previous studies, the same mineral supplement was shown to substantially increase desmosome formation along the lateral surface of adjacent epithelial cells in colon organoid culture without a major effect on tight junctions (22–24). Thus, while functioning tight junctions are directly responsible for permeability control, our past findings suggest that permeability control cannot be optimally maintained in a mechanically active tissue such as the colon when cell-cell cohesion (22–24) is disrupted. The data presented here extend this conclusion to cell-matrix interactions. These data suggest that cell-matrix interactions also play a contributing role in barrier function. It should be noted, of course, that cell-matrix interactions are complex and involve multiple cell surface and cytoskeletal proteins on the one hand, and several different matrix moieties on the other. The use of a single blocking antibody which could interfere, presumably, with laminin binding to many different partners precludes a more precise determination of the relative importance of different individual cell-matrix protein combinations to the overall result. This notion has been tested before by targeting a specific molecule (kalinin) using an antibody in keratinocyte cell culture, and concluding that that kalinin (i.e., laminin 332) is the critical component of basement membrane (48).

How Aquamin® functions to improve barrier structure/function is not fully understood. Many of the matrix-related proteins found to be up-regulated here are products of differentiation, and calcium, the most abundant mineral in the algae product (21), is the quintessential regulator of epithelial differentiation (49). While calcium is undoubtedly critical, many of the additional trace elements in Aquamin® have a higher affinity than calcium itself for the extracellular calcium-sensing receptor (50–52). They act like calcimimetic agents to “left-shift” the response to calcium. We believe that increasing the elaboration of critical barrier proteins is an important mechanism by which Aquamin® promotes barrier function. At the same time, calcium, magnesium and manganese are all critical to the protein-protein interactions that mediate cell-cell and cell-matrix adhesion interactions (53). Thus, the contribution of Aquamin® to barrier function likely extends beyond driving new protein production.

The studies carried out here made use of a sophisticated *ex vivo* culture system (colon tissue maintained in organoid culture) and comprise data from seven different subjects for proteomics, three different subjects for organoid-derived monolayer culture on transwell membrane and three different subjects for the tissue cohesion assay. This *ex vivo* system used here may have its limitation while lacking *in vivo* environment, but it provides a good substitute for colonic tissue to test interventions *ex vivo* (54). Still, whether the effects obtained *in vitro* have relevance to what occurs *in vivo* remains to be demonstrated. In an effort to begin addressing this issue, we have recently carried out a pilot phase trial in which 10 healthy subjects were treated with the same multi-mineral product (Aquamin®) used here. To summarize the results of this pilot study, there were no tolerability issues with daily Aquamin® ingestion over a 90-day period and no safety concerns (55, 56). Equally important, when Aquamin®-treated subjects were compared to subjects receiving placebo for the same period, we saw up-regulation of laminin chains along with increased levels of other basement membrane components and hemidesmosome moieties in colonic biopsies (56). Subjects receiving calcium alone (i.e., the most abundant mineral in Aquamin®) also demonstrated increases in several of the same molecules, but the degree of up-regulation with calcium alone was lower than that seen with Aquamin® (56).

As a follow-up, we are conducting a 180-day interventional trial with Aquamin® in UC patients (ClinicalTrials.gov: NCT03869905). In addition to evaluating therapeutic benefit, the same approaches used in the earlier trial with healthy individuals (immunohistology and proteomics) are being used to evaluate proteins changes in the colon over the course of intervention. In parallel, the urine lactulose/mannitol ratio (57) is being assessed to provide a direct measure of treatment effects on gastrointestinal permeability (ClinicalTrials.gov: NCT04855799). If successful, Aquamin® or a similarly formulated product could be used as a low-cost, low- to no-toxicity adjuvant therapy to improve gastrointestinal barrier function in individuals suffering from a variety of gastrointestinal maladies. At the very least, individuals with barrier defect-associated gastrointestinal conditions should be encouraged to include an adequate source of calcium and other minerals in their diet. Unfortunately, deficiencies in calcium and other critical mineral components are widespread throughout the world (58) and this is especially true for those consuming a Western-style diet (59, 60).

Finally, there is another group of diseases—epidermolysis bullosa and related conditions—that are manifestations of mutations in various basement membrane, desmosomal/hemidesmosomal and keratin genes (61). At the same time, there are case reports and studies that provide evidence of an association between bullous pemphigoid and inflammatory bowel disease (62–64). At this point, we can only speculate as to whether optimizing the expression of multiple cell-cell and cell-matrix adhesion molecules in an individual might overcome, at least in part, the consequences of a function-modifying mutation in one or another critical component. If this turns out to be the case, it could open the door to a new adjuvant therapeutic approach. While speculative for now, experimental models in which a hypothesis could be tested are available (65–67).

In summary, an intact barrier is required for healthy gastrointestinal function. While cell-cell adhesion structures are well-known participants in effective barrier function, the present study provides evidence that cell-matrix interactions are also important. These studies show, furthermore, that a multi-mineral natural product has the capacity to stimulate the production of cell-matrix adhesion moieties and, concomitantly, to improve barrier control.

## Data Availability Statement

The mass spectrometry proteomics datasets presented in this study can be found in online repositories – on ProteomeXchange Consortium (PRIDE partner repository) with identifier PXD020244 (for UC-derived organoids) and identifier PXD026923 (for normal colon organoids).

## Ethics Statement

The studies involving human participants were reviewed and approved by Institutional Review Board at the University of Michigan Medical School (IRBMED). The patients/participants provided their written informed consent to participate in this study.

## Author Contributions

MA and JV: conceptualization, resources, writing—original draft preparation, and funding acquisition. MA, SM, and JV: methodology, validation, investigation, writing—review and editing. MA and SM: software, formal analysis, and data curation. MA: visualization, supervision, and project administration. All authors have read and agreed to this version of the manuscript.

## Funding

This study was supported by the National Institutes of Health (NIH) grant CA201782 including supplemental funding through the Office of Dietary Supplements to JV and by an MCubed (University of Michigan) grant to MA.

## Conflict of Interest

The authors declare that the research was conducted in the absence of any commercial or financial relationships that could be construed as a potential conflict of interest.

## Publisher's Note

All claims expressed in this article are solely those of the authors and do not necessarily represent those of their affiliated organizations, or those of the publisher, the editors and the reviewers. Any product that may be evaluated in this article, or claim that may be made by its manufacturer, is not guaranteed or endorsed by the publisher.

## Supplementary Material

Supplementary Data Sheet 1: S1 File Mineral composition of Aquamin® Soluble.

Supplementary Data Sheet 2: Figure 1 Protein-Protein interactions of proteins shown in Figure 1.

Supplementary Data Sheet 3: Figure 2 Source documentation for Western blot data shown in Figure 2.

Supplementary Table 1 GO-based STRING enrichment data.

## References

[1] Salim SY Söderholm JD. Importance of disrupted intestinal barrier in inflammatory bowel diseases. Inflamm Bowel Dis. (2011) 17:362-81. 10.1002/ibd.21403 20725949

[2] Antoni L Nuding S Wehkamp J Stange EF. Intestinal barrier in inflammatory bowel disease. World J Gastroenterol. (2014) 20:1165-79. 10.3748/wjg.v20.i5.1165 24574793 PMC3921501

[3] Lee JY Wasinger VC Yau YY Chuang E Yajnik V Leong RW. Molecular pathophysiology of epithelial barrier dysfunction in inflammatory bowel diseases. Proteomes. (2018) 6:17. 10.3390/proteomes6020017 29614738 PMC6027334

[4] Vivinus-Nébot M Frin-Mathy G Bzioueche H Dainese R Bernard G Anty R. Functional bowel symptoms in quiescent inflammatory bowel diseases: role of epithelial barrier disruption and low-grade inflammation. Gut. (2014) 63:744-52. 10.1136/gutjnl-2012-304066 23878165

[5] Pearson AD Eastham EJ Laker MF Craft AW Nelson R. Intestinal permeability in children with Crohn's disease and coeliac disease. Br Med J. (1982) 285:20-1. 10.1136/bmj.285.6334.20 6805795 PMC1499105

[6] Luissint AC Parkos CA Nusrat A. Inflammation and the intestinal barrier: leukocyte-epithelial cell interactions, cell junction remodeling, and mucosal repair. Gastroenterology. (2016) 151:616-32. 10.1053/j.gastro.2016.07.008 27436072 PMC5317033

[7] Dunlop SP Hebden J Campbell E Naesdal J Olbe L Perkins AC. Abnormal intestinal permeability in subgroups of diarrhea-predominant irritable bowel syndromes. Am J Gastroenterol. (2006) 101:1288-94. 10.1111/j.1572-0241.2006.00672.x 16771951

[8] Flügel A Schulze-Koops H Heesemann J Kühn K Sorokin L Burkhardt H. Interaction of enteropathogenic Yersinia enterocolitica with complex basement membranes and the extracellular matrix proteins collagen type IV, laminin-1 and−2, and nidogen/entactin. J Biol Chem. (1994) 269:29732-8. 10.1016/S0021-9258(18)43942-7 7961965

[9] Moreira AP Texeira TF Ferreira AB Peluzio Mdo C Alfenas Rde C. Influence of a high-fat diet on gut microbiota, intestinal permeability and metabolic endotoxaemia. Br J Nutr. (2012) 108:801-9. 10.1017/S0007114512001213 22717075

[10] Thaiss CA Levy M Grosheva I Zheng D Soffer E Blacher E. Hyperglycemia drives intestinal barrier dysfunction and risk for enteric infection. Science. (2018) 359:1376-83. 10.1126/science.aar3318 29519916

[11] Meddings JB Swain MG. Environmental stress-induced gastrointestinal permeability is mediated by endogenous glucocorticoids in the rat. Gastroenterology. (2000) 119:1019-28. 10.1053/gast.2000.18152 11040188

[12] Clayburgh DR Shen L Turner JR. A porous defense: the leaky epithelial barrier in intestinal disease. Lab Invest. (2004) 84:282-91. 10.1038/labinvest.3700050 14767487

[13] Turner JR. Molecular basis of epithelial barrier regulation: from basic mechanisms to clinical application. Am J Pathol. (2006) 169:1901-9. 10.2353/ajpath.2006.060681 17148655 PMC1762492

[14] Shen L Su L Turner JR. Mechanisms and functional implications of intestinal barrier defects. Dig Dis. (2009) 27:443-9. 10.1159/000233282 19897958 PMC2814011

[15] Turner JR. Intestinal mucosal barrier function in health and disease. Nat Rev Immunol. (2009) 9:799-809. 10.1038/nri2653 19855405

[16] Camilleri M Madsen K Spiller R Greenwood-Van Meerveld B Verne GN. Intestinal barrier function in health and gastrointestinal disease. Neurogastroenterol Motil. (2012) 24:503-12. 10.1111/j.1365-2982.2012.01921.x 22583600 PMC5595063

[17] Cereijido M Valdés J Shoshani L Contreras RG. Role of tight junctions in establishing and maintaining cell polarity. Annu Rev Physiol. (1998) 60:161-77. 10.1146/annurev.physiol.60.1.161 9558459

[18] Aijaz S Balda MS Matter K. Tight junctions: molecular architecture and function. Int Rev Cytol. (2006) 248:261-98. 10.1016/S0074-7696(06)48005-0 16487793

[19] Green KJ Simpson CL. Desmosomes: new perspectives on a classic. J Invest Dermatol. (2007) 127:2499-515. 10.1038/sj.jid.5701015 17934502

[20] Kowalczyk AP Green KJ. Structure, function, and regulation of desmosomes. Prog Mol Biol Transl Sci. (2013) 116:95-118. 10.1016/B978-0-12-394311-8.00005-4 23481192 PMC4336551

[21] Adey WH McKibbin DL. Studies on the maerl species Phymatolithon calcareum (Pallas) nov. comb. and Lithothamnium corallioides Crouan in the Ria de Vigo. Botanical Marina. (1970) 13:100–6. 10.1515/botm.1970.13.2.100

[22] Attili D McClintock SD Rizvi AH Pandya S Rehman H Nadeem DM. Calcium-induced differentiation in normal human colonoid cultures: Cell-cell / cell-matrix adhesion, barrier formation and tissue integrity. PLoS ONE. (2019) 14:e0215122. 10.1371/journal.pone.0215122 30995271 PMC6469792

[23] McClintock SD Attili D Dame MK Richter A Silvestri SS Berner MM. Differentiation of human colon tissue in culture: effects of calcium on trans-epithelial electrical resistance and tissue cohesive properties. PLoS ONE. (2020) 15:e0222058. 10.1371/journal.pone.0222058 32134920 PMC7058309

[24] Aslam MN McClintock SD Attili D Pandya S Rehman H Nadeem DM. Ulcerative colitis-derived colonoid culture: a multi-mineral-approach to improve barrier protein expression. Front Cell Dev Biol. (2020) 8:577221. 10.3389/fcell.2020.577221 33330453 PMC7719760

[25] Schmehl K Florian S Jacobasch G Salomon A Körber J. Deficiency of epithelial basement membrane laminin in ulcerative colitis affected human colonic mucosa. Int J Colorectal Dis. (2000) 15:39-48. 10.1007/s003840050006 10766090

[26] Verbeke S Gotteland M Fernández M Bremer J Ríos G Brunser O. Basement membrane and connective tissue proteins in intestinal mucosa of patients with coeliac disease. J Clin Pathol. (2002) 55:440-5. 10.1136/jcp.55.6.440 12037027 PMC1769663

[27] Bouatrouss Y Herring-Gillam FE Gosselin J Poisson J Beaulieu JF. Altered expression of laminins in Crohn's disease small intestinal mucosa. Am J Pathol. (2000) 156:45-50. 10.1016/S0002-9440(10)64704-9 10623652 PMC1868644

[28] Spenlé C Lefebvre O Lacroute J Méchine-Neuville A Barreau F Blottière HM. The laminin response in inflammatory bowel disease: protection or malignancy? PLoS ONE. (2014) 9:e111336. 10.1371/journal.pone.0111336 25347196 PMC4210184

[29] Timpl R Tisi D Talts JF Andac Z Sasaki T Hohenester E. Structure and function of laminin LG modules. Matrix Biol. (2000) 19:309-17. 10.1016/S0945-053X(00)00072-X 10963991

[30] Colognato H Yurchenco PD. Form and function: the laminin family of heterotrimers. Dev Dyn. (2000) 218:213-34. 10.1002/(SICI)1097-0177(200006)218:2<213::AID-DVDY1>3.0.CO;2-R 10842354

[31] Turck N Gross I Gendry P Stutzmann J Freund JN Kedinger M. Laminin isoforms: biological roles and effects on the intracellular distribution of nuclear proteins in intestinal epithelial cells. Exp Cell Res. (2005) 303:494-503. 10.1016/j.yexcr.2004.10.025 15652360

[32] Gonzales M Haan K Baker SE Fitchmun M Todorov I Weitzman S. A cell signal pathway involving laminin-5, alpha3beta1 integrin, and mitogen-activated protein kinase can regulate epithelial cell proliferation. Mol Biol Cell. (1999) 10:259-70. 10.1091/mbc.10.2.259 9950675 PMC25167

[33] Miyoshi H Stappenbeck TS. *In vitro* expansion and genetic modification of gastrointestinal stem cells in spheroid culture. Nat Protoc. (2013) 8:2471-82. 10.1038/nprot.2013.153 24232249 PMC3969856

[34] Zou WY Blutt SE Crawford SE Ettayebi K Zeng XL Saxena K. Human intestinal enteroids: new models to study gastrointestinal virus infections. Methods Mol Biol. (2019) 1576:229-47. 10.1007/7651_2017_1 28361480 PMC5752619

[35] Fabregat A Sidiropoulos K Viteri G Forner O Marin-Garcia P Arnau V. Reactome pathway analysis: a high-performance in-memory approach. BMC Bioinformatics. (2017) 18:142. 10.1186/s12859-017-1559-2 28249561 PMC5333408

[36] Sekiguchi R Yamada KM. Basement membranes in development and disease. Curr Top Dev Biol. (2018) 130:143-91. 10.1016/bs.ctdb.2018.02.005 29853176 PMC6701859

[37] Pozzi A Yurchenco PD Iozzo RV. The nature and biology of basement membranes. Matrix Biol. (2017) 57-58:1-11. 10.1016/j.matbio.2016.12.009 28040522 PMC5387862

[38] Baker SE Hopkinson SB Fitchmun M Andreason GL Frasier F Plopper G. Laminin-5 and hemidesmosomes: role of the alpha 3 chain subunit in hemidesmosome stability and assembly. J Cell Sci. (1996) 109:2509-20. 10.1242/jcs.109.10.2509 8923212

[39] Green KJ Jones JC. Desmosomes and hemidesmosomes: structure and function of molecular components. FASEB J. (1996) 10:871-81. 10.1096/fasebj.10.8.8666164 8666164

[40] Chidgey M. Plakin Proteins, Hemidesmosomes and Human Disease. In: eLS editor. John Wiley & Sons (2012). 10.1002/9780470015902.a0024527

[41] Has C. Hemidesmosomes: how much plakins do they need? Exp Dermatol. (2016) 25:263-4. 10.1111/exd.12939 26740080

[42] Polari L Alam CM Nyström JH Heikkilä T Tayyab M Baghestani S. Keratin intermediate filaments in the colon: guardians of epithelial homeostasis. Int J Biochem Cell Biol. (2020) 129:105878. 10.1016/j.biocel.2020.105878 33152513

[43] Zupancic T Stojan J Lane EB Komel R Bedina-Zavec A Liovic M. Intestinal cell barrier function in vitro is severely compromised by keratin 8 and 18 mutations identified in patients with inflammatory bowel disease. PLoS ONE. (2014) 9:e99398. 10.1371/journal.pone.0099398 24915158 PMC4051775

[44] Corfe BM Majumdar D Assadsangabi A Marsh AM Cross SS Connolly JB. Inflammation decreases keratin level in ulcerative colitis; inadequate restoration associates with increased risk of colitis-associated cancer. BMJ Open Gastroenterol. (2015) 2:e000024. 10.1136/bmjgast-2014-000024 26462276 PMC4599170

[45] Nishiuchi R Takagi J Hayashi M Ido H Yagi Y Sanzen N. Ligand-binding specificities of laminin-binding integrins: a comprehensive survey of laminin-integrin interactions using recombinant alpha3beta1, alpha6beta1, alpha7beta1 and alpha6beta4 integrins. Matrix Biol. (2006) 25:189-97. 10.1016/j.matbio.2005.12.001 16413178

[46] De Arcangelis A Hamade H Alpy F Normand S Bruyère E Lefebvre O. Hemidesmosome integrity protects the colon against colitis and colorectal cancer. Gut. (2017) 66:1748-60. 10.1136/gutjnl-2015-310847 27371534 PMC5595104

[47] Stutzmann J Bellissent-Waydelich A Fontao L Launay JF Simon-Assmann P. Adhesion complexes implicated in intestinal epithelial cell-matrix interactions. Microsc Res Tech. (2000) 51:179-90. 10.1002/1097-0029(20001015)51:2<179::AID-JEMT9>3.0.CO;2-4 11054868

[48] Rousselle P Lunstrum GP Keene DR Burgeson RE. Kalinin: an epithelium-specific basement membrane adhesion molecule that is a component of anchoring filaments. J Cell Biol. (1991) 114:567-76. 10.1083/jcb.114.3.567 1860885 PMC2289097

[49] Hennings H Holbrook KA. Calcium regulation of cell-cell contact and differentiation of epidermal cells in culture. An ultrastructural study. Exp Cell Res. (1983) 143:127-42. 10.1016/0014-4827(83)90115-5 6186504

[50] Huang Y Zhou Y Castiblanco A Yang W Brown EM Yang JJ. Multiple Ca(2+)-binding sites in the extracellular domain of the Ca(2+)-sensing receptor corresponding to cooperative Ca(2+) response. Biochemistry. (2009) 48:388-98. 10.1021/bi8014604 19102677 PMC2627791

[51] Singh N Aslam MN Varani J Chakrabarty S. Induction of calcium sensing receptor in human colon cancer cells by calcium, vitamin D and aquamin: promotion of a more differentiated, less malignant and indolent phenotype. Mol Carcinog. (2015) 54:543-53. 10.1002/mc.22123 26076051

[52] Carrillo-López N Fernández-Martín JL Alvarez-Hernández D González-Suárez I Castro-Santos P Román-García P. Lanthanum activates calcium-sensing receptor and enhances sensitivity to calcium. Nephrol Dial Transplant. (2010) 25:2930-7. 10.1093/ndt/gfq124 20233740

[53] Tiwari S Askari JA Humphries MJ Bulleid NJ. Divalent cations regulate the folding and activation status of integrins during their intracellular trafficking. J Cell Sci. (2011) 124:1672-80. 10.1242/jcs.084483 21511727 PMC3085436

[54] Varani J McClintock SD Aslam MN. Organoid culture to study epithelial cell differentiation and barrier formation in the colon: bridging the gap between monolayer cell culture and human subject research. In Vitro Cell Dev Biol Anim. (2021) 57:174-90. 10.1007/s11626-020-00534-6 33403624 PMC8720467

[55] Aslam MN Bassis CM Bergin IL Knuver K Zick SM Sen A. A calcium-rich multimineral intervention to modulate colonic microbial communities and metabolomic profiles in humans: results from a 90-day trial. Cancer Prev Res. (2020) 13:101-16. 10.1158/1940-6207.CAPR-19-0325 31771942 PMC7528938

[56] Aslam MN McClintock SD Jawad-Makki MAH Knuver K Ahmad HM Basrur V. A multi-mineral intervention to modulate colonic mucosal protein profile: results from a 90-day trial in human subjects. Nutrients. (2021) 13:939. 10.3390/nu13030939 33799486 PMC8002192

[57] Sequeira IR Lentle RG Kruger MC Hurst RD. Standardising the lactulose mannitol test of gut permeability to minimise error and promote comparability. PLoS ONE. (2014) 9:e99256. 10.1371/journal.pone.0099256 24901524 PMC4047110

[58] Balk EM Adam GP Langberg VN Earley A Clark P Ebeling PR. International osteoporosis foundation calcium steering committee. global dietary calcium intake among adults: a systematic review. Osteoporos Int. (2017) 28:3315-24. 10.1007/s00198-017-4230-x 29026938 PMC5684325

[59] Aslam MN Varani J. The western-style diet, calcium deficiency and chronic disease. J Nutr Food Sci. (2016) 6:3. 10.4172/2155-9600.1000496

[60] U.S. Department of Health and Human Services; U.S. Department of Agriculture. 2015-−2020 Dietary Guidelines for Americans. 8th ed. Washington, DC: U.S. Department of Health and Human Services (2015).

[61] Fine JD Eady RA Bauer EA Bauer JW Bruckner-Tuderman L Heagerty A. The classification of inherited epidermolysis bullosa (EB): report of the third international consensus meeting on diagnosis and classification of EB. J Am Acad Dermatol. (2008) 58:931-50. 10.1016/j.jaad.2008.02.004 18374450

[62] Sachsenberg-Studer EM Runne U Wehrmann T Wolter M Kriener S Engels K. Bullous colon lesions in a patient with bullous pemphigoid. Gastrointest Endosc. (2001) 54:104-8. 10.1067/mge.2001.115472 11427857

[63] Seo JW Park J Lee J Kim MY Choi HJ Jeong HJ. A case of pemphigus vulgaris associated with ulcerative colitis. Intest Res. (2018) 16:147-50. 10.5217/ir.2018.16.1.147 29422810 PMC5797262

[64] Chen YJ Juan CK Chang YT Wu CY Ho HJ Tseng HC. Association between inflammatory bowel disease and bullous pemphigoid: a population-based case-control study. Sci Rep. (2020) 10:12727. 10.1038/s41598-020-69475-0 32728039 PMC7391771

[65] Natsuga K Shinkuma S Nishie W Shimizu H. Animal models of epidermolysis bullosa. Dermatol Clin. (2010) 28:137-42. 10.1016/j.det.2009.10.016 19945627

[66] Bruckner-Tuderman L McGrath JA Robinson EC Uitto J. Animal models of epidermolysis bullosa: update 2010. J Invest Dermatol. (2010) 130:1485-8. 10.1038/jid.2010.75 20463671

[67] Heimbach L Li N Diaz A Liu Z. Experimental animal models of bullous pemphigoid. G Ital Dermatol Venereol. (2009) 144:423-31. 19755945

