# Supplementary material for: Cell-Matrix Interactions Contribute to Barrier Function in Human Colon Organoids
Source: Front Med (Lausanne). 2022 Mar 10;9:838975. doi: 10.3389/fmed.2022.838975 (PMC8960989; doi:10.3389/fmed.2022.838975)
Supplement: Supplementary Data Sheet 2: Figure 1 — Protein-Protein interactions of proteins shown in Figure 1. [file Data_Sheet_2.pdf]

Supplementary Material

1.1 Supplementary Fig 1.

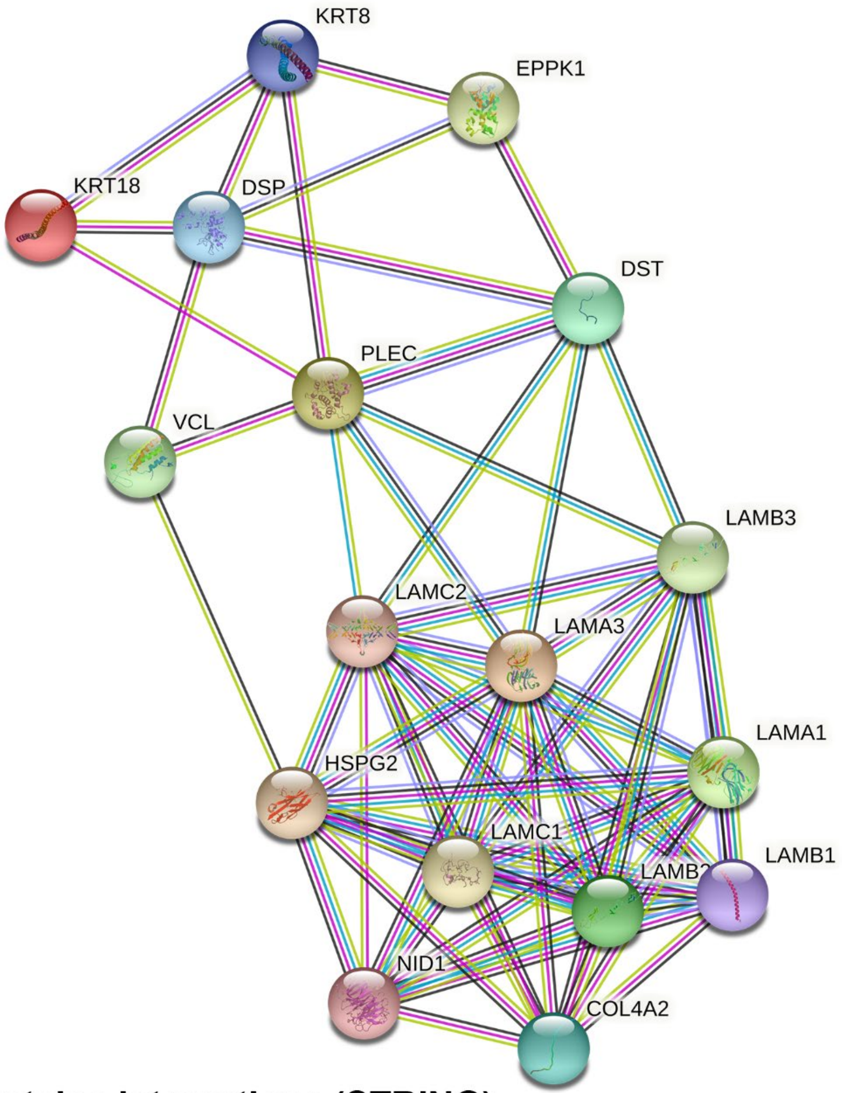

Supplementary Fig 1: Protein-Protein interactions (PPI) of proteins shown in Fig 1.

There are strong protein-protein interactions, and a tight cluster is visible comprising laminin subunits, COL4A2, perlecan and Nidogen. The protein-protein interaction (PPI) enrichment p-value was $< 1.0 \times 10^{-16}$.
