# Supplementary material for: Cell-Matrix Interactions Contribute to Barrier Function in Human Colon Organoids
Source: Front Med (Lausanne). 2022 Mar 10;9:838975. doi: 10.3389/fmed.2022.838975 (PMC8960989; doi:10.3389/fmed.2022.838975)
Supplement: Supplementary Data Sheet 3: Figure 2 — Source documentation for Western blot data shown in Figure 2. [file Data_Sheet_3.pdf]

# *Supplementary Material*

### 1.2.1 Supplementary Fig 2.

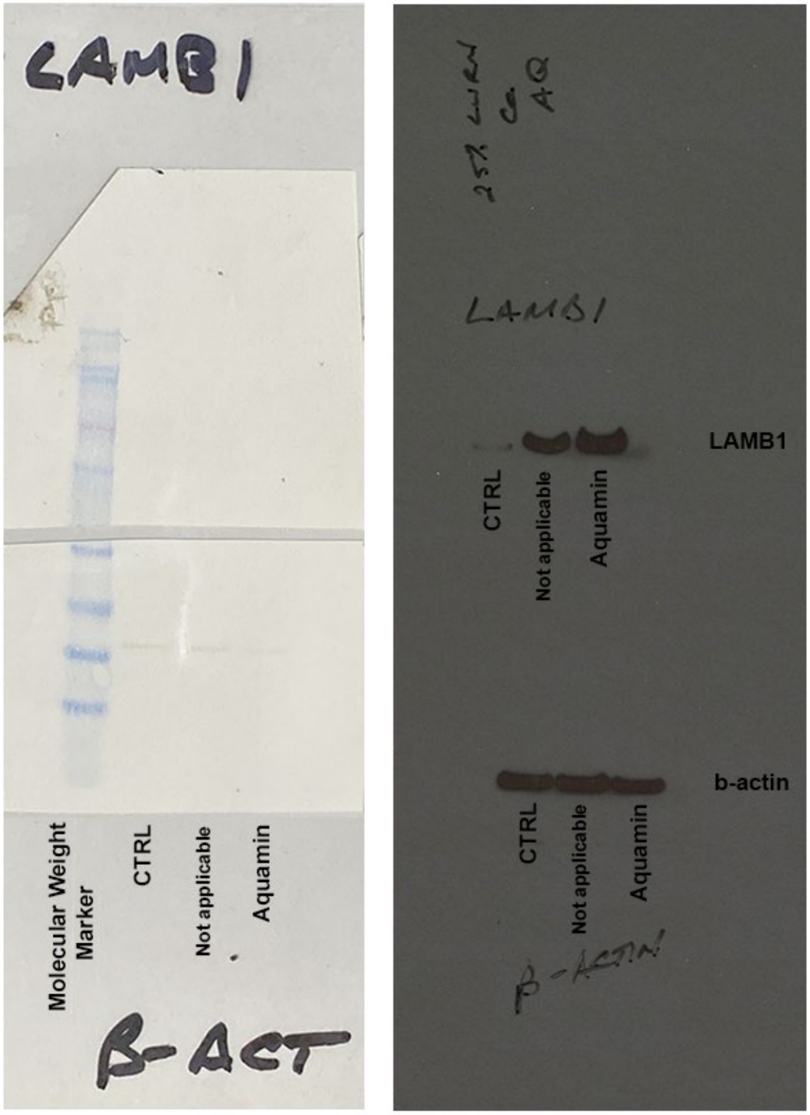


**Supplementary Fig 2: Source documentation for Western blot data shown in Fig 2:** The nitrocellulose sheets (top and bottom) along with the film generated from each nitrocellulose sheet are shown together. Laminin β1 protein was probed by Western blotting and presented in Figure 2 (in

the manuscript). β-actin was used as a loading control. Hand-written notes are original and typed-up labels are for presentation purposes.

Procedure: After completion of the resistance measurements on Day 3, organoids derived monolayer cell lysates were prepared and separated by gel electrophoresis using pre-cast gels. Following this, the proteins were transferred to a nitrocellulose membrane in the standard manner. This was done in a single electrophoresis run. Following completion of electrophoresis and protein transfer, the nitrocellulose membrane was cut horizontally into a top and bottom portion knowing the expected location of the bands of these two proteins based on their respective molecular weights (198kDa for LAMβ1 and 42 kDa for β-actin). The appropriate antibodies were used to probe for the proteins of interest. These membranes were processed and developed on a single film to obtain images. SuperSignal WestPico Plus (34577; Thermo Scientific) detection reagent was used, and bands were visualized by exposing the membranes on CL-XPosure Film (34090; Thermo Scientific). Developing was performed using Konica Minolta SRX-101A.

There were three conditions (lanes) in this experiment. These are labeled and presented in the final composite figure as lane 1: Control and lane 3: Aquamin. [Note: a sample unrelated to the present study was included between the control and Aquamin lands in the original blot and included here for completeness. It is not included in the presentation figure (Fig 2 of the manuscript).
